# Bioaccessibility and Dynamic Changes in Free and Bound Phenolics in Rice Bean (*Vigna umbellata*) During Simulated Digestion

**DOI:** 10.3390/foods15111985

**Published:** 2026-06-03

**Authors:** Xiao Peng, Qinzhang Jiang, Jucai Xu, Yanxian Feng, Rihui Wu, Ruili Yang, Wu Li

**Affiliations:** 1 School of Pharmacy and Food Engineering, Wuyi University, Jiangmen 529020, China; 15898546702@163.com (X.P.); yanxianfeng@wyu.edu.cn (Y.F.); wyuchemwrh@126.com (R.W.); 2 Key Laboratory of Food Nutrition and Functional Food of Hainan Province, College of Food Science and Engineering, Hainan University, Haikou 570228, China; jiang3149@126.com; 3 College of Food Science, South China Agricultural University, Guangzhou 510642, China; rlyang77@scau.edu.cn

**Keywords:** rice beans, polyphenols, bound polyphenols, digestion, bioaccessibility

## Abstract

This study investigated the dynamic changes and bioaccessibility of free and bound phenolics in rice bean (*Vigna umbellata*) during simulated gastrointestinal digestion. A total of 34 phenolic compounds were identified and quantified across oral, gastric, and intestinal phases by *UPLC-MS/MS* detected. Gastric digestion was identified as the critical stage for phenolic release, with multiple flavonoids increasing 2–3-fold, including rutin (205%), isoquercitrin (226%), and procyanidin B1 (134%). In contrast, in the intestinal phase, flavonoids including procyanidin B1, epicatechin, and quercetin became undetectable after extensive degradation, while phenolic acids such as *p*-hydroxybenzoic acid (157%) and trans-cinnamic acid (200%) accumulated gradually. Phloroglucinol showed a progressive accumulation increased continuously during digestion (10 to 36 mg/kg DW). Most bound phenolics remained remarkably stable, with over 85% retained throughout upper gastrointestinal transit, except for bound *p*-coumaric acid and phloroglucinol, which were gradually released. Notably, 3,4-dihydroxyphenylacetic acid was detected only in the bound form across all phases. These findings reveal the dual fates of rice bean phenolics, especially the bound fraction, and underscore the importance of their release and transformation during digestion when evaluating the bioactivity of rice bean polyphenols.

## 1. Introduction

Rice bean (*Vigna umbellata*) is a widely cultivated edible legume in Southeast Asia, valued as both a food source and traditional medicine. Previous studies have demonstrated that rice beans are particularly rich in polyphenolic compounds, including phenolic acids (e.g., caffeic acid, protocatechuic acid, benzoic acid) and flavonoids (e.g., procyanidin B1, epicatechin, isoquercitrin), which have been associated with antioxidant, anti-inflammatory, and antidiabetic activities [1,2]. Our previous research revealed that approximately 70% of phenolics in rice bean exist in bound form, mainly comprising phenolic acids and flavonoid glycosides [3]. However, despite these compositional insights, the digestive release patterns, transformation characteristics, and subsequent bioaccessibility of rice bean phenolic compounds, particularly the dominant bound fraction, remain largely unexplored. Given that polyphenols constitute the primary functional components of rice bean and are predominantly present in the bound form, elucidating their dynamic changes during gastrointestinal digestion and subsequent bioaccessibility is essential for accurately assessing the nutritional and health value of this legume.

Dietary polyphenols, a diverse group of secondary metabolites abundant in plant-based foods, have garnered increasing attention for their potential role in reducing the risk of chronic diseases, including cardiovascular ailments, certain cancers, and neurodegenerative disorders [4,5]. Extensive research has demonstrated that these health benefits are closely associated with their biological activities, such as antioxidant and anti-inflammatory properties [6]. However, a critical factor governing polyphenols’ *in vivo* efficacy is their bioavailability, which exhibits remarkable variability among different polyphenols. For instance, the absolute bioavailability of epigallocatechin gallate (EGCG) and resveratrol is typically less than 1–5%, largely due to extensive metabolism in the intestine and liver [7]. In contrast, simpler phenolic acids such as gallic acid exhibit relatively higher bioavailability [8]. This discrepancy highlights that the biological efficacy of polyphenols is not solely determined by their native content in foods but is critically dependent on their fate during gastrointestinal digestion, including release from the food matrix, structural transformation, and subsequent absorption [9].

Yao et al. quantified the contents of total phenols and total flavonoids in rice beans of different cultivars [10]. Mass spectrometry identified *p*-coumaric acid, quercetin and other polyphenols with antioxidant activity in the extract. Sangsukiam et al. investigated the release of phenolic compounds from thermally preprocessed rice beans during *in vitro* simulated digestion, measured the total phenolic content of the digested products, and identified five polyphenols including catechin and vitexin [11]. Li et al. studied the dynamic changes in total phenols and total flavonoids in powdered rice bean samples during simulated digestion [12]. Given most polyphenols in rice beans exist in the bound form, it is necessary to characterize the composition and dynamic changes in free and bound polyphenols throughout simulated digestion, and further elucidate their bioaccessibility to explore the nutritional potential of rice bean polyphenols during digestion. However, apart from the detection of free phenols, the research of the composition and dynamic changes in free and bound polyphenols in rice beans during the digestion process has not been reported.

Fruits, vegetables, and grains are major sources of dietary polyphenols. Although the per-unit polyphenol content of grains may be lower than that of certain fruits, their high daily consumption makes them principal contributors, accounting for an estimated 40–50% of total dietary polyphenol intake [13]. A defining feature of grain polyphenols is that a substantial proportion exists not as free compounds, but as bound phenolics covalently linked to cell wall macromolecules. Studies have shown that approximately 70–85% of polyphenols in maize and wheat are present as bound phenolics, while this proportion reaches 60–70% in legumes [14]. The release of bound phenolics depends on hydrolysis by digestive enzymes or fermentation by the colonic microbiota to become bioaccessible. Crucially, gastrointestinal digestion of polyphenols is not merely a process of release, but one of dynamic transformation. During transit through the oral, gastric, and intestinal environments, phenolic compounds undergo structural modifications that fundamentally alter their composition [15]. The extent and nature of these transformations vary among different polyphenols, contributing to the marked differences in their bioavailability. Therefore, understanding changes in phenolic composition throughout digestion is essential for evaluating the health potential of grain-based foods. Therefore, this study aimed to investigate the changes and bioaccessibility of both free and bound polyphenols throughout a simulated gastrointestinal digestion process. By tracking their compositional evolution across the oral, gastric, and small intestinal phases, we sought to provide a theoretical basis for understanding the digestive behavior and functional evaluation of rice bean polyphenols.

## 2. Materials and Methods

### 2.1. Experimental Material

A single harvest season rice beans, were only obtained from the agricultural products transfer base in Zhaoqing City, Guangdong Province (Produced in Zhaoqing, China, 2022).

### 2.2. Reagents

Gallic acid, quercetin, caffeic acid, ellagic acid, catechol, naringin, protocatechuic acid, ferulic acid, syringic acid, rutin, isoquercitrin, epicatechin, catechin, gallocatechin, daidzin, daidzein, vitexin, isovitexin, genistin, genistein, taxafolin, morin, sinapic acid, glycitein, 3,4-dihydroxyphenylacetic acid, trans-cinnamic acid, benzoic acid, phloroglucinol, *p*-coumaric acid and *p*-hydroxybenzoic acid were LC-MS grade, above 98% (Yuanye Bio-Technology Co., Ltd., Shanghai, China). Kaempferol-3-O-rutinoside, kaempferol, procyanidin B1 and procyanidin B2 were LC-MS grade, above 98% (Pusi Biotechnology Co., Ltd., Chengdu, China). EDTA-2Na(Ethylenediaminetetraacetic acid disodium salt), fluorescein, 2,20-azinobis (3-ethylbenzothiazoline-6-sulfonic acid (ABTS), 1,1-diphenyl-2-picryl- hydrazyl (DPPH), (±)-6-Hydroxy-2,5,7,8-tetram- ethylchromane-2-carboxylic acid(Trolox), 2,4,6tri(pyridin-2-yl)-1,3,5-triazine (TPTZ), 2,2′-azobis (2-amidinopropane) dihydrochloride (AAPH) were purchased from Aladdin Biochemical Technology Co., Ltd. (Shanghai, China). Thermo Fisher Scientific (Waltham, MA, USA) provided the methanol (100%, HPLC quality), formic acid (99.9%, LC-MS grade), and acetonitrile (100%, LC-MS grade). Pepsin (3000 U/g, from pig), was purchased from Sigma Chemical Co., (St. Louis, MO, USA). Pancreatic enzymes (300 U/g, from porcine), was purchased from Aladdin. Bile salts (from porcine), α-amylase (1500 U/mL), KCl (99%), KH_2_PO_4_ (99%), NaHCO_3_ (99%), MgCl_2_·6H_2_O (99%), (NH_4_)_2_CO_3_ (99%) were purchased from Macklin Biochemical Technology Co., Ltd. (Shanghai, China).

### 2.3. Plant Materials Collection and Preparation

Mature rice beans with uniform size, intact shape, and no visible signs of mold were selected. The rice beans were ground into powder using a Chinese herbal medicine grinder (2500 A, SuFeng, Yongkang, China), then sieved through 60-mesh and 80-mesh standard test sieves to obtain powder with particle size ranging from 0.18 mm to 0.25 mm. Oversized coarse particles and undersized fine powder were discarded, accounting for approximately 12% of the total ground material in weight. The resulting powder was used directly for the experiment.

### 2.4. Digestion Process

The digestion method was referred to reference [16]. All digestion steps were performed in 50 mL polypropylene centrifuge tubes. For the oral digestion phase, preparation of the simulated oral digestive fluid, 15.1 mL of 15.1 mM KCl, 3.7 mL of 3.7 mM KH_2_PO_4_, 6.8 mL of 13.6 mM NaHCO_3_, 0.5 mL of 0.15 mM MgCl_2_·6H_2_O, and 0.06 mL of 0.06 mM (NH_4_)_2_CO_3_ were mixed, and adjusted to 500 mL with ultrapure water (≥18.2 MΩ·cm). 0.5 g of rice bean powder was mixed with 2.1 mL of simulated oral digestive fluid, followed by the addition of 0.3 mL of α-amylase (1500 U/mL), 15 μL of 3 mM CaCl_2_, and 2.085 mL of ultrapure water. The mixture was incubated in a shaking incubator (orbital shaker, 20 mm amplitude) at 37 °C in the dark at 95 rpm for 5 min.

For the gastric digestion phase, the orally digested sample was mixed with 4.5 mL of simulated gastric digestive fluid (pH = 2.0), followed by 0.96 mL of pepsin (1500 U/mL) and 3 µL of 3 mM CaCl_2_. The mixture was incubated in a circular orbital shaking incubator with an orbital amplitude of 20 mm at 37 °C in the dark at 95 rpm for 2 h. For the small intestinal digestion phase, to the sample after gastric digestion, 13.2 mL of simulated intestinal digestive fluid (pH = 7.0) was added, together with 3 mL of trypsin (800 U/mL), 1.5 mL of bile salts, and 24 µL of 24 mM CaCl_2_. The mixture was incubated in a shaking incubator at 37 °C in the dark at 200 rpm for 2 h.

After each digestion phase, the sample was centrifuged at 9000 rpm for 10 min in room temp (25 °C). The supernatant (digestive fluid) was collected and evaporated to dryness at 35 °C, then re-dissolved in 3.0 mL of 80% methanol. The precipitate was retained as the digestion residue. Free phenols and bound phenols were extracted with 80% (*v*/*v*) methanol and alkaline hydrolysis, respectively. The polyphenol content in the digestive fluid was calculated as, total polyphenol content in the re-dissolved digestive fluid relative to the dry weight of the original rice bean sample before digestion.

### 2.5. The Extraction of Polyphenols

For the extraction of free phenols, organic solvent extraction methods were used. The extraction was carried out according to the method in the reference [17]. 0.5 g of the sample (dry weight) was weighed, 15.0 mL of 80% methanol (containing 1% (*v*/*v*) formic acid) was added, and ultrasonic treatment was performed at 360 W ultrasonic frequency of 40 kHz for 30 min, and the extraction temperature was kept constant at 25 °C throughout the process. The residue was sequentially extracted three times by centrifugation, collected each time, and the three supernatants were combined. The pooled supernatant was then evaporated to dryness via rotary evaporation at 45 °C, and the resulting dry residue was reconstituted with 80% methanol to a final volume of 5.0 mL.

For bound phenol extraction, the alkaline hydrolysis method was used. The method was based on Tang et al. [18]. The residue after free phenol extraction was dried to constant weight by rotary evaporation. 0.5 g of the residue after alcohol extraction was added with 5.0 mL of NaOH (10 M) and 12.0 mL of EDTA-2Na solution (0.5 M). Following hydrolysis in a shaking water bath (30 °C, 4 h), the reaction was quenched by acidification to pH 2.0 using 6 M HCl. After centrifugation (9000 rpm, 10 min), the supernatant was collected and subjected to liquid–liquid extraction with ethyl acetate (1:1, *v*/*v*) in triplicate. The organic layers were combined, concentrated via rotary evaporation at 45 °C, and brought to a final volume of 5.0 mL with 80% methanol.

### 2.6. Determination of Total Phenolic Content

The total phenol content was determined according to the Folin-phenol method reported in the article [19]. Take 0.125 mL of the polyphenol extract, diluted to an appropriate concentration within the linear range of the calibration curve by ultrapure water, add 0.5 mL of water and 0.125 mL of Folin–Ciocalteu reagent, mix thoroughly, and allow the mixture to stand for 6 min. Add 1.25 mL of 70 g/L Na_2_CO_3_ solution and 1.0 mL of water. Incubate at 30 °C in the dark for 90 min, take 0.2 mL and transfer it to a round-bottom type 96-well microplate, and measure the absorbance at 760 nm by a Microplate reader (BioTek Synergy Neo2). In the experiment, gallic acid was used as the standard, with a concentration range of 0.01–0.1 mg/mL. The total phenolic content was expressed as milligrams of gallic acid equivalents per gram of dry weight (mg GAE/g DW).

The total flavonoid content was determined according to the previously reported aluminum chloride method [20], with some modifications. Add 0.1 mL of methanol to 0.1 mL of the sample, mix with 0.05 mL of 50 g/L NaNO_2_ solution for 6 min; add 0.05 mL of 100 g/L AlCl_3_ solution and mix evenly, react for 6 min; finally, add 0.4 mL of NaOH (1 M) and 0.3 mL of methanol to stop the reaction and incubate at room temperature for 15 min; measure the absorbance at 510 nm after the incubation is completed. Rutin (0.05–0.6 mg/mL) was used as the standard curve. The total flavonoid content was expressed as milligrams of rutin equivalents per gram of dry weight (mg RE/g DW).

### 2.7. Antioxidant Activity Assays

ABTS stock solution: 176 µL of potassium persulfate solution (140 mM) was mixed thoroughly with 10 mL of ABTS solution (7 mM). The mixture was incubated in the dark at room temperature for 16 h to obtain the ABTS stock solution. DPPH working solution: 0.0197 g of DPPH was dissolved and diluted to 500 mL with methanol to obtain a 100 µmol/L DPPH working solution. FRAP working solution: Acetate buffer solution (300 mM, pH = 3.6), TPTZ solution (10 mM), and FeCl_3_ solution (20 mM) were mixed at a volume ratio of 10:1:1. The mixture was pre-incubated in a water bath at 37 °C before use. The FRAP working solution was prepared fresh immediately before use. ORAC reagents: PBS buffer (pH 7.4) was prepared using 0.1 mol/L sodium dihydrogen phosphate and disodium hydrogen phosphate. The PBS buffer was then used to prepare fluorescein sodium solution (150 nmol/L) and AAPH solution (119.4 mmol/L).

The ABTS free radical scavenging ability test was conducted according to the method [21]. The ABTS stock solution was diluted with water to an absorbance of 0.7 ± 0.02 at 734 nm, and then 0.1 mL of the sample was mixed with 2.0 mL of the diluted ABTS solution at room temperature in the dark for 6 min. The absorbance was measured at 734 nm. Trolox, a water-soluble vitamin E analog (different from tocopherol and tocotrienol), was employed as the standard control at concentrations ranging from 0.01 to 0.15 μg/mL. The experimental results were expressed in µmol TE/g DW.

The DPPH free radical scavenging was determined according to the previous report [22]. In simple terms, 0.05 mL of the sample reacted with 0.4 mL of DPPH (0.1 mmol/L) at room temperature for 30 min, and the absorbance was measured at 517 nm. Water-soluble vitamin E (0.01–0.15 mg/mL) was used as the standard curve. The experimental results were expressed in µmol TE/g DW.

The FRAP antioxidant activity was determined following the method, with minor modifications [23]. 0.03 mL of the sample was incubated with 0.9 mL of FRAP working solution at room temperature in the dark for 30 min, and the absorbance was measured at 593 nm. The standard curve was composed of sodium acetate buffer (300 mM), TPTZ (10 mM), and different concentrations of ferrous sulfate solutions (0.1–1.5 mmol/L) in a volume ratio of 10:1:1. The experimental results were expressed in µmol Fe (II)SE/g DW.

The assay for the oxygen radical absorbance capacity (ORAC) was based on a previously described procedure [24], with minor modifications. 0.025 mL of the sample and 0.1 mL of 0.15 µmol/L fluorescein sodium working solution in pH 7.4 phosphate-buffered saline (PBS) were added to a black 96-well plate and equilibrated at 37 °C for 20 min, with intermittent shaking. Then, 0.075 mL of AAPH (119.4 mM) solution prepared with pH 7.4 phosphate-buffered saline (PBS) was added to the system, and the fluorescence intensity was measured using a multifunctional microplate reader every 3.5 min at the excitation wavelength of 485 nm and the emission wavelength of 535 nm for 35 times. The area under the fluorescence decay curve was integrated to determine the fluorescence burst area. Trolox (6.25–500 μmol/mL) was used as the standard curve, and the results were expressed in µmol TE/g DW.

### 2.8. UPLC-MS/MS Analysis

The phenolic compounds in rice beans were identified using a UPLC-Q-Exactive Orbitrap-MS/MS system equipped with an electrospray ionization (ESI) source (Thermo Fisher Scientific, Shanghai, China). The ultra-high performance liquid chromatography conditions were as follows. Chromatographic column: Waters ACQUITY UPLC BEH C18 column (2.1 × 100 mm, 1.7 μm); mobile phase A was 0.1% formic acid water, B was acetonitrile. The gradient elution program was: 0–3 min 95–85% A, 3–11 min 85–70% A, 11–15 min 70–50% A, 15–21 min 50–10% A, 21–22 min 10–95% A. Each sample was injected before maintaining for 3 min, with a flow rate of 0.15 mL/min, column temperature of 40 °C, and injection volume of 2.0 µL.

The mass spectrometry conditions were as follows: spray voltage maintained at 3.2 kV, capillary temperature 320 °C, sheath gas pressure 35 arb (arbitrary units, relative flow), auxiliary gas pressure 10 arb. Positive and negative ion scans were used to record mass spectrometry information. The resolution was 7000, the scan range was 70–1050 *m*/*z*, and the automatic gain control entered the number of ions in the orbitrap (AGC target) for compounds without standard samples: primary ions 3 × 10^6^, secondary ions 1 × 10^5^, maximum injection time: primary ions 100 ms, secondary ions 50 ms.

Polyphenols were mainly identified by comparing the chromatographic and retention times of the samples with those of the standards. The compounds without standards were identified by the accurate mass (M-H) of the parent ion, typical mass spectrometry fragmentation patterns and references. The quantification of polyphenols was carried out by establishing an external standard curve with standards.

### 2.9. Data Analysis

All experiments were performed in triplicate. Data were expressed as mean ± standard deviation (SD). Statistical analysis was conducted using SPSS 26 software. Graphical visualization, data standardization, principal component analysis (PCA), including data included, averaging, centering, normalization and Pearson correlation analysis were performed using Origin 2021b. Differences among groups were evaluated by one-way analysis of variance (ANOVA), with *p* < 0.05 considered statistically significant.

## 3. Results and Discussion

### 3.1. Phenolic Content

**Figure 1:**
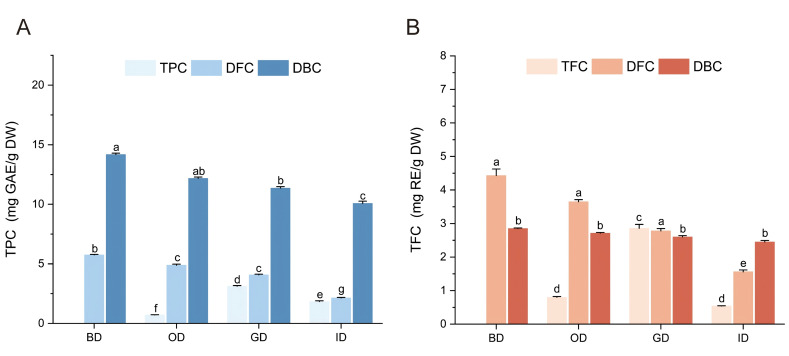
The changes in total phenols and total flavonoids during the simulated digestion process. (**A**) The total phenol content in the simulated digestion process of rice beans. (**B**) The total flavonoid content in the simulated digestion process of rice beans. BD: before digestion. OD: oral digestion. GD: gastric digestion. ID: intestinal digestion. TPC: Total phenol content in the digestion liquid. TFC: Total flavonoid content in the digestion liquid. DFC: Free phenolic content in the digestion residue. DBC: Bound phenolic content in the digestion residue. Different letters indicate significant differences in the content of digestive fluid at different stages, with *p* < 0.05.

The changes in the total phenolic and total flavonoid contents of rice bean during simulated in vitro gastrointestinal digestion are shown in Figure 1A,B. Before digestion, the contents of free and bound phenolics were 5.8 and 14 mg GAE/g DW, respectively; the contents of free and bound flavonoids were 4.4 and 2.9 mg RE/g DW, respectively. In the digestive fluids, the total phenolic contents in the oral, gastric, and small intestinal phases were 0.71, 3.1, and 1.8 mg GAE/g DW, respectively, and the total flavonoid contents were 0.80, 2.9, and 0.54 mg RE/g DW, respectively. Further analysis of the phenolic fractions in the digestive residues showed that the free phenolic contents were 4.9, 4.1, and 2.1 mg GAE/g DW, respectively; the bound phenolic contents were 12, 11, and 10 mg GAE/g DW, respectively; the free flavonoid contents were 3.7, 2.8, and 1.6 mg RE/g DW, respectively; and the bound flavonoid contents were 2.7, 2.6, and 2.5 mg RE/g DW, respectively.

The contents of polyphenols and flavonoids in the digestive fluid of the oral phase were the lowest. This initial decrease cannot be attributed to degradation or release, given the short duration and mild conditions of oral digestion. Instead, it may reflect the rapid formation of complexes between free phenolic substances and salivary proteins and starch, generating non-extractable non-covalent complexes. This phenomenon is consistent with previous research findings that proline-rich salivary proteins have a high affinity for polyphenols, particularly proanthocyanidins and flavonols, leading to precipitation and reduced extractability [25].

Compared to pre-digestion, approximately 54% and 65% of the total phenolics and total flavonoids were already released in the stomach, with the highest content of both found in the gastric digestive fluid. The stomach is the primary site for polyphenol release during the digestion of rice beans. In addition to the longer digestion time compared to the oral phase, this may be related to the acidic environment of the stomach (pH 2.0). Such low pH conditions can partially hydrolyze glycosidic bonds and disrupt the non-covalent interactions between phenolic compounds and cell wall polysaccharides, thereby facilitating their transfer into the aqueous phase. Consequently, the gastric phase is a critical site for the initial release of phenolic compounds from the food matrix, a finding consistent with the established “acidic extraction” effect observed in various plant-based foods [26].

During the small intestinal phase, the content of total phenolics released into the digestive fluid decreased compared to that in the gastric phase, whereas the bound phenolic fractions remained remarkably stable throughout the entire digestion process. The reduction rates of bound phenolics and bound flavonoids were above 83% and 90%, respectively. This differential behavior underscores the distinction between the two phenolic fractions: free phenolics are susceptible to environmental fluctuations and matrix interactions, while bound phenolics, covalently linked to cell wall macromolecules, exhibit inherent resistance to digestive enzymes and pH changes. The stability of bound phenolics in rice bean was notably higher than that observed in other legumes, such as soybean and black bean [27,28], and aligned with the behavior of certain bound phenolics in coffee dietary fiber, where only about 17% of bound phenolics were released during gastric and intestinal digestion [29]. This exceptional stability may be attributed to the high content of proanthocyanidins and condensed tannins in the seed coat of rice bean, which form robust complexes with dietary fiber.

Our results showed that 0.71–3.1 mg GAE/g DW of free phenolics were released during digestion. The low bioaccessibility of free phenolic compounds in the upper gastrointestinal tract suggests that their direct absorption in the small intestine is limited. In contrast, the unreleased free phenolics (2.1 mg GAE/g DW in the digestive residue) and the bound phenolic fractions resisted upper gastrointestinal digestion but may be available for colonic metabolism, potentially contributing to delayed and sustained health benefits.

### 3.2. Phenolic Composition

As shown in Table 1, free and bound phenolics released during the simulated digestion were characterized by comparison with standards, MS/MS fragments, and literature data. A total of 34 phenolic compounds were identified using authentic standards.

A total of 28, 29, and 22 polyphenols compounds were identified in the oral (OJ), gastric (GJ), and intestinal (IJ) digestive fluids of rice bean, respectively (Table 1). As shown in Figure 1A, the results showed that phloroglucinol was newly detected during the oral digestion stage compared to pre-digestion samples. This suggests that even the brief oral phase with α-amylase activity is sufficient to initiate limited hydrolysis of ester bonds and glycosidic linkages, releasing small phenolic acids and flavonoid aglycone precursors [30]. In addition, Jakobek et al. [15] reported that chlorogenic acid in apple peel underwent isomerization during the simulated salivary phase, indicating that phenolic transformations can occur rapidly upon exposure to salivary electrolytes and enzymes.

Compared with the oral phase, syringic acid and daidzein were newly detected in the gastric fluid (GJ), while free caffeic acid, free daidzin and glycitein were no longer detected as free phenolic compounds in the gastric residue (GF). The disappearance of free daidzin and the simultaneous appearance of its aglycone form, daidzein, in gastric fluid suggests that daidzin may be hydrolyzed by gastric acid or pepsin to release its aglycone. The disappearance of caffeic acid from GF, while it was detected in GJ, suggests that a small portion was released into the digestive fluid, but the majority may have undergone degradation or transformation. Liu et al. [30] identified multiple degradation products of caffeic acid during the intestinal phase, including protocatechuic acid, *p*-hydroxybenzoic acid, and ferulic acid, formed through oxidation and hydroxyl displacement. Although their study focused on the intestinal phase, similar transformations may begin under gastric conditions. The absence of glycitein and catechol from GF, despite their presence in OF, may indicate their participation in oxidation or polymerization reactions under gastric conditions.

Compared with the gastric stage, multiple compounds were no longer detected in IJ, including caffeic acid, syringic acid, procyanidin B1, procyanidin B2, epicatechin, quercetin and catechol. In the small intestinal residue free phenolic fraction (IF), 23 polyphenolic compounds were detected. Compared with GF, epicatechin, isovitexin, daidzein, morin, glycitein, and genistein were not detected in IF, indicating extensive degradation or metabolic transformation. The disappearance of these compounds can be attributed to the neutral-to-alkaline intestinal environment, which promotes oxidation, polymerization, and structural rearrangement of flavonoids. Liu et al. [31] identified multiple degradation products of phenolic acids in the intestinal phase, formed through oxidation, hydroxyl displacement, and dehydration. Salas-Millán et al. [32] further demonstrated that acylated flavonoids exhibited a decrease during intestinal digestion.

Throughout the entire simulated digestion process, distinct distribution patterns emerged. 3,4-dihydroxyphenylacetic acid and naringenin existed exclusively in bound form across all phases. Notably, caffeic acid, epicatechin, and daidzein, which were present in the free phenolic fraction of undigested rice beans, were only detected in bound form after entering the small intestine stage. This suggests that these compounds likely underwent degradation or transformation during digestion. Liu et al. [31] demonstrated that caffeic acid undergoes extensive degradation in the intestinal phase, generating multiple products including protocatechuic acid, *p*-hydroxybenzoic acid, and ferulic acid through oxidation and hydroxyl displacement. Phloroglucinol, absent in pre-digestion samples, appeared in oral fluid and progressively accumulated throughout digestion, providing indirect evidence for flavonoid A-ring degradation. The stage-specific emergence and disappearance of phenolic compounds in digestion demonstrate that gastrointestinal digestion is not merely a release process but a dynamic transformation cascade. These findings underscore the importance of considering both release and transformation when evaluating the potential health effects of dietary polyphenols.

### 3.3. Contents Individual Phenolic Compounds

As shown in Table 2, the release and content of monomeric phenols exhibited pronounced stage-specificity during simulated digestion of rice beans. Total quantified phenolic content in digestive fluid peaked at the gastric stage before declining in the intestinal stage, reflecting enhanced release under acidic conditions followed by degradation or transformation in the neutral intestinal environment.

Compared with the oral stage, gastric digestion significantly increased the release of multiple phenolic compounds, including gallic acid (increased by 132%, from 1.8 to 4.1 mg/kg DW), *p*-hydroxybenzoic acid (increased by 124%, from 1.2 to 2.6 mg/kg DW), ellagic acid (increased by 199%, from 1.5 to 4.6 mg/kg DW), benzoic acid (increased by 66%, from 4.1 to 6.7 mg/kg DW), procyanidin B1 (increased by134%, from 17 to 39 mg/kg DW), rutin (increased by 205%, from 18 to 54 mg/kg DW), isoquercitrin (increased by 226%, from 19 to 62 mg/kg DW), and quercetin (increased by 180%, from 2.3 to 6.5 mg/kg DW). This 2- to 3-fold increase in multiple flavonoids during gastric digestion indicates that the acidic gastric environment and digestive enzymes may hydrolyze cell wall polysaccharides or glycosidic bonds, promoting the release of bound phenolics and the degradation/transformation of polyphenols. Studies by Jakobek et al. [15] and Alves et al. [33] have also reported that diverse phenolic compounds embedded in food matrices are extensively released or transformed during the gastric digestion phase.

Upon transition to the small intestinal phase, profound compositional changes occurred. Caffeic acid, procyanidin B1, procyanidin B2, epicatechin, and quercetin became undetectable in digestive fluid, while rutin decreased by 75% (from 54 to 14 mg/kg DW) and isoquercitrin decreased by 81% (from 62 to 12 mg/kg DW). Concurrently, several phenolic acids accumulated: protocatechuic acid (from 3.5 to 4.2 mg/kg DW), *p*-hydroxybenzoic acid (from 2.6 to 6.7 mg/kg DW), ferulic acid (from 1.0 to 1.2 mg/kg DW), and trans-cinnamic acid (from 0.79 to 2.4 mg/kg DW). Notably, phloroglucinol showed progressive accumulation from 10.0 (oral) to 24 (gastric) to 36 mg/kg DW (intestinal) in the digestive fluid. This reciprocal relationship between flavonoid disappearance and phenolic acid accumulation suggests that flavonoids are catabolized via C-ring fission under alkaline intestinal conditions. The accumulation of phloroglucinol is particularly significant, as the characteristic A-ring degradation product of flavonoids, its progressive increase concomitant with the disappearance of proanthocyanidins and flavonoid glycosides strongly suggests that flavonoids undergo C-ring fission during gastrointestinal digestion [34,35].

Analysis of the digestive residue fractions revealed complementary dynamics. Free phenolics in the residue progressively decreased throughout digestion, with cumulative reductions exceeding 70% for procyanidin B1 (from 161 to 11 mg/kg DW), rutin (from 65 to 15 mg/kg DW), isoquercitrin (from 177 to 53 mg/kg DW), and quercetin (from 20 to 3.4 mg/kg DW). This continuous decline confirms that free phenolics are steadily released from the food matrix into the digestive fluid, though their subsequent detection depends on their stability under prevailing conditions.

In contrast, bound phenolics in the residue exhibited remarkable stability. From the oral to intestinal phase, most bound phenolics showed minimal changes, such as gallic acid, protocatechuic acid, taxafolin and 3,4-dihydroxyphenylacetic acid. This stability aligns with findings from legume digestion studies, where bound phenolic acids are not significantly released during digestive phases due to their covalent ester and ether linkages to cell wall polysaccharides, which resist human digestive enzymes [36]. However, our results also showed that bound phloroglucinol and *p*-coumaric acid progressively decreased during digestion, with a concomitant increase in their concentrations in the digestive fluid. Notably, Phloroglucinol in the bound fraction decreased from 42 to 28 mg/kg DW, while its content significantly increased in both the digestive fluid and the free residue fraction during digestion. These patterns indicate that bound phloroglucinol and *p*-coumaric acid were gradually liberated from the bound fraction during digestion, releasing into digestive fluid as free forms.

The quantitative data collectively demonstrate that the digestive fate of rice bean bound phenolics follows distinct patterns: the majority remain stably associated with the insoluble matrix throughout upper gastrointestinal digestion, while a small subset, particularly phloroglucinol and p-coumaric acid, exhibit gradual release. Previous studies have shown that only a fraction of phenolic compounds in legumes is bioaccessible in the upper gastrointestinal tract, with the majority remaining associated with the insoluble matrix [36]. Our results show that more than 85% of bound phenolics in rice beans survive transit through the stomach and small intestinal, entering the colon intact where they become substrates for microbial fermentation [37].

During intestinal digestion, the bioaccessibility of individual phenolic compounds in rice bean varied markedly and was classified into four levels, calculate according to formula bioaccessibility (%) = (phenolic content detected in digestive fluid/total phenolic content in the sample) × 100. Trans-cinnamic acid (224%), ferulic acid (200%), benzoic acid (184%), *p*-hydroxybenzoic acid (170%) and p-coumaric acid (122%) exceeded 100%, attributed to sustained release of bound phenolics, structural conversion, and enhanced extractability. Protocatechuic acid (62%) and kaempferol-3-O-rutinoside (56%) showed moderate bioaccessibility. Low bioaccessibility was observed for rutin (23.15%), ellagic acid (14%), taxifolin (7.0%), catechin (5.9%), gallocatechin (7.6%), isoquercitrin (7.2%), and gallic acid (5.9%) due to degradation, oxidation, or strong matrix binding. Caffeic acid, procyanidin B1, B2, epicatechin, and quercetin were undetectable, indicating complete degradation. Overall, phenolic acids had higher bioaccessibility than flavonoids [38].

### 3.4. Principal Component Analysis

**Figure 2:**
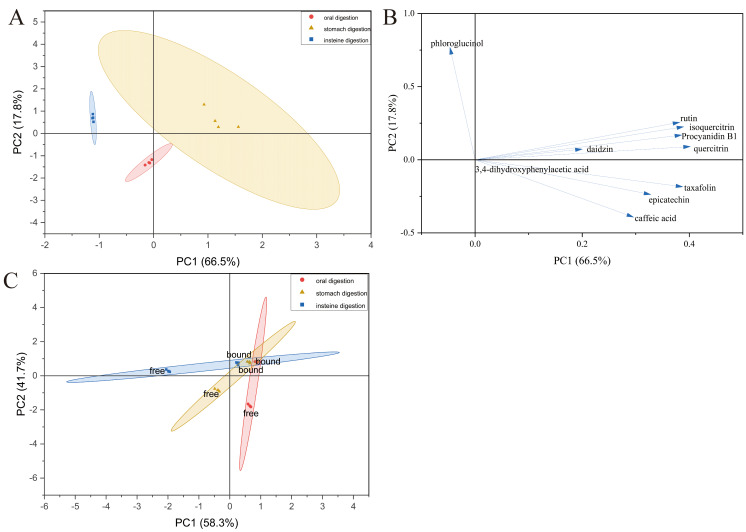
PCA plot of the phenolic changes in rice beans under different digestive stages. (**A**) PCA score plot of individual phenolic compounds in the digestive liquid phase; (**B**) Loading plot identifying the major phenolic compounds contributing to the separation in (**A**); (**C**) PCA score plot of free and bound phenolic fractions in the digestive residue phase.

Principal component analysis (PCA) was employed to evaluate differences in the distribution patterns of phenolic compounds during simulated gastrointestinal digestion. As shown in Figure 2A, PCA of individual phenolics detected in digestive fluids revealed that principal component 1 (PC1) and principal component 2 (PC2) accounted for 67% and 18% of total variance, respectively, cumulatively explaining 85% of total variation. Samples from the three digestive phases formed three clearly distinguishable clusters with no overlap, confirming that each digestive stage imparts a unique chemical signature on the phenolic profile. The majority of individual phenolics clustered closely around the gastric digestion phase, indicating that the stomach is a critical stage driving the release and transformation of rice bean polyphenols. The synchronously elevated content of most phenolics in gastric fluid are consistent with the role of the acidic environment in disrupting phenolic-matrix interactions and promoting solubilization. In contrast, a few compounds located distant from this cluster, such as 3,4-dihydroxyphenylacetic acid, exhibited unique stability, consistent with their exclusive presence in the bound fraction throughout digestion.

In Figure 2B, free and bound phenolic fractions in digestive residues were clearly separated, indicating significant compositional differences across digestive phases. These results demonstrate that the digestive process fundamentally alters both the existing forms and the composition of rice bean polyphenols, with the bound fraction maintaining distinct stability while the free fraction undergoes dynamic transformation. The distinct clustering patterns further highlight the differential contributions of free and bound fractions to the overall phenolic profile during digestion.

## 4. Conclusions

This study elucidated the digestive fate of free and bound phenolics in rice bean (*Vigna umbellata*) during simulated gastrointestinal digestion. The stomach was identified as the primary site for phenolic release, with flavonoids (rutin, isoquercitrin) and glycoside hydrolysis (daidzin to daidzein) being extensively liberated. The neutral-to-alkaline environment of the intestinal phase induced marked degradation and/or transformation of multiple flavonoids, including the disappearance of procyanidin B1, procyanidin B2, epicatechin, and quercetin. Phloroglucinol continuously accumulated throughout digestion, increasing from 10 to 36 mg/kg DW. In contrast, most bound phenolics remained stable, except for bound p-coumaric acid and phloroglucinol, which exhibited gradual release during digestion. Notably, 3,4-dihydroxyphenylacetic acid were detected exclusively in the bound form across all digestive phases. The bound phenolic fraction was confirmed as the primary contributor to overall antioxidant capacity (Figure S1). These findings reveal that rice bean bound phenolics largely survive gastric and small intestinal digestion, whereas rice bean free phenolics undergo extensive release and subsequent degradation or transformation. This study provides a theoretical basis for understanding the digestive fate of rice bean polyphenols and for optimizing processing strategies to modulate their bioaccessibility.

## Figures and Tables

**Table 1:** Composition of polyphenols in rice bean digestive fluids and residue.

Number	RT (min)	Phenolics	Formula	△m (ppm)	*m*/*z* [M-H]	*m*/*z* Fragments	Before Digest		Oral			Gastric			Intestinal		
							DF	DB	OJ	OF	OB	GJ	GF	GB	IJ	IF	IB
	Phenolic acids																
1	2.97	gallic acid	C_7_H_6_O_5_	−1.8	169.0134	125.0232	+	+	+	+	+	+	+	+	+	+	+
2	4.98	protocatechuic acid	C_7_H_6_O_4_	−3.9	153.0182	109.0281	+	+	+	+	+	+	+	+	+	+	+
3	6.47	*p*-hydroxybenzoic acid	C_7_H_6_O_3_	−2.2	137.0232	93.0332	+	+	+	+	+	+	+	+	+	+	+
4	7.1	caffeic acid	C_9_H_8_O_4_	−2.2	179.0340	135.0439	+	+	+	+	+	+	−	+	−	−	+
5	7.21	syringic acid	C_9_H_10_O_5_	−1	197.0448	182.0216, 166.9979	+	+	−	+	−	+	+	−	−	+	−
6	8.89	ellagic acid	C_14_H_6_O_8_	−0.3	300.9988	257.0087	+	+	+	+	+	+	+	+	+	+	+
7	8.97	*p*-coumaric acid	C_9_H_8_O_3_	−3.1	163.0390	119.0489	+	+	+	+	+	+	+	+	+	+	+
8	9.52	sinapic acid	C_11_H_12_O_5_	−0.9	223.0605	208.0370, 193.0132	+	+	+	+	+	+	+	+	+	+	+
9	9.67	ferulic acid	C_10_H_10_O_4_	−1.6	193.0498	178.0263, 149.0597	+	+	+	+	+	+	+	+	+	+	+
10	11.9	benzoic acid	C_7_H_6_O_2_	−5.8	121.0283	94.0283, 122.0319	+	+	+	+	+	+	+	+	+	+	+
11	15.62	trans-cinnamic acid	C_9_H_8_O_2_	−3.4	147.0441	102.9474	+	+	+	+	+	+	+	+	+	+	+
	Flavonoids																
12	4.42	gallocatechin	C_15_H_14_O_7_	0	305.0667	261.0774, 179.0339	+	+	+	+	+	+	+	+	+	+	+
13	5.55	procyanidin B1	C_30_H_26_O_12_	0	577.1351	451.1034, 407.0771	+	+	+	+	−	+	+	−	−	+	−
14	6.28	catechin	C_15_H_14_O_6_	0.3	289.0718	245.0814, 179.0340	+	+	+	+	+	+	+	+	+	+	+
15	6.61	procyanidin B2	C_30_H_26_O_12_	0	577.1351	451.1053, 407.0773	+	+	+	+	+	+	+	+	−	+	+
16	7.24	epicatechin	C_15_H_14_O_6_	0.3	289.0718	245.0815, 179.0341	+	+	+	+	+	+	+	+	−	−	+
17	7.81	daidzin	C_21_H_20_O_9_	0	415.1035	253.0506	+	+	−	+	+	−	−	+	−	−	+
18	8.84	rutin	C_27_H_30_O_16_	0	609.1465	300.0275	+	+	+	+	+	+	+	+	+	+	+
19	8.95	isovitexin	C_21_H_20_O_10_	−0.2	431.0983	341.0665, 311.0561	+	−	+	+	−	+	+	−	+	−	−
20	9.06	vitexin	C_21_H_20_O_10_	−0.7	431.0981	311.0560, 341.0664	+	+	+	+	−	+	+	−	+	+	−
21	9.35	isoquercitrin	C_21_H_20_O_12_	0.2	463.0883	300.0273, 301.0350	+	+	+	+	+	+	+	+	+	+	+
22	9.81	genistin	C_21_H_20_O_10_	0.5	431.0986	269.0446, 268.0378	+	−	+	+	−	+	+	−	+	+	−
23	9.93	kaempferol-3-O-rutinoside	C_27_H_30_O_15_	0	593.1517	285.0403	+	+	+	+	+	+	+	+	+	+	+
24	10.07	taxifolin	C_15_H_12_O_7_	+1.0	303.0508	259.0714, 241.0615	+	+	+	+	+	+	+	+	+	+	+
25	13.19	morin	C_15_H_10_O_7_	2.3	301.0356	273.0402, 255.0301	+	−	−	−	−	−	+	−	−	−	−
26	13.53	daidzein	C_15_H_10_O_4_	0	253.0506	135.0087, 107.1326	+	−	−	+	−	+	+	−	+	−	−
27	13.96	glycitein	C_16_H_12_O_5_	0	283.0607	268.0374	+	−	+	+	−	+	−	−	−	−	−
28	14.53	quercetin	C_15_H_10_O_7_	1.7	301.0354	178.9978, 151.0026	+	−	+	+	+	+	+	+	−	+	+
29	16.13	genistein	C_15_H_10_O_5_	1.1	269.0458	133.0282	+	−	+	+	−	−	+	−	−	−	−
30	16.19	naringenin	C_15_H_12_O_5_	−0.4	271.0611	151.0025, 119.0489	−	+	−	−	+	−	−	+	−	−	+
31	16.39	kaempferol	C_15_H_10_O_6_	2.5	285.0406	257.0461	+	+	+	+	+	+	+	+	+	+	+
	Other phenols																
32	2.82	phloroglucinol	C_6_H_6_O_3_	+0.8	127.0391	109.0287	−	+	+	+	+	+	+	+	+	+	+
33	5.49	3,4-dihydroxyphenylacetic acid	C_8_H_8_O_4_	−1.2	167.0342	123.0438	−	+	−	−	+	−	−	+	−	−	+
34	6.71	catechol	C_6_H_6_O_2_	−8.3	109.0281	91.0175	+	−	+	+	−	+	−	−	−	−	−

All compounds were identified in [M−H]^−^ mode, except for number 32 was detected in [M+H]^+^ mode. DF: before digestive free phenol; DB: before digestive bound phenol; OJ: Oral digestive fluid; OF: free phenol from oral residue; OB: oral residue bound phenol; GJ: gastric digestive fluid; GF: gastric residue free phenol; GB: gastric residue bound phenol; IJ: small intestinal digestive fluid; IF: small intestinal residue free phenol; IB: small intestinal residue bound phenol; + indicates detected; − indicates no detected.

**Table 2:** Content of phenolic compounds in rice bean simulated gastrointestinal digestion (mg/kg DW).

Number	Phenolics	OJ/OF/OB	GJ/GF/GB	IJ/IF/IB
1	gallic acid	1.8 ± 0.21 ^B^	4.1 ± 0.31 ^A^	0.16 ± 0.03 ^C^
		4.1 ± 0.17 ^a^	1.8 ± 0.10 ^b^	0.54 ± 0.02 ^c^
		24 ± 1.6 ^a^	24 ± 2.5 ^a^	23 ± 0.67 ^a^
2	protocatechuic acid	3.1 ± 0.17 ^C^	3.5 ± 0.53 ^B^	4.2 ± 0.34 ^A^
		7.1 ± 0.57 ^a^	3.1 ± 0.14 ^b^	2.9 ± 0.16 ^b^
		29 ± 1.9 ^a^	28 ± 3.2 ^a^	26 ± 2.4 ^a^
3	*p*-hydroxybenzoic acid	1.2 ± 0.14 ^C^	2.6 ± 0.19 ^B^	6.7 ± 0.26 ^A^
		5.9 ± 0.21 ^a^	1.7 ± 0.10 ^c^	4.3 ± 0.14 ^b^
		51 ± 2.6 ^a^	44 ± 3.1 ^ab^	49 ± 3 ^a^
4	caffeic acid	0.02 ± 0.01 ^A^	0.02 ± 0.01 ^A^	-
		0.06 ± 0.04 ^a^	-	-
		0.29 ± 0.01 ^a^	0.14 ± 0.03 ^b^	0.05 ± 0.01 ^c^
6	ellagic acid	1.5 ± 0.21 ^B^	4.6 ± 0.14 ^A^	1.1 ± 0.08 ^C^
		9.3 ± 0.19 ^b^	5.2 ± 0.26 ^c^	12 ± 0.23 ^a^
		3.0 ± 0.28 ^a^	2.3 ± 0.33 ^b^	1.9 ± 0.25 ^b^
7	*p*-coumaric acid	1.8 ± 0.11 ^B^	0.93 ± 0.04 ^C^	1.9 ± 0.21 ^A^
		2.0 ± 0.11 ^a^	0.80 ± 0.08 ^b^	0.12 ± 0.03 ^c^
		4.5 ± 0.22 ^a^	2.9 ± 0.19 ^b^	1.1 ± 0.23 ^c^
8	erucic acid	0.82 ± 0.03 ^B^	0.80 ± 0.05 ^B^	0.88 ± 0.09 ^A^
		0.81 ± 0.09 ^a^	0.33 ± 0.03 ^b^	0.07 ± 0.01 ^c^
		0.61 ± 0.06 ^a^	0.47 ± 0.04 ^b^	0.27 ± 0.02 ^c^
9	ferulic acid	0.73 ± 0.01 ^C^	0.95 ± 0.22 ^B^	1.2 ± 0.08 ^A^
		0.92 ± 0.08 ^a^	0.38 ± 0.05 ^b^	0.08 ± 0.01 ^c^
		0.49 ± 0.08 ^a^	0.44 ± 0.04 ^a^	0.43 ± 0.03 ^a^
10	benzoic acid	4.1 ± 0.37 ^B^	6.7 ±1.75 ^A^	3.1 ± 0.39 ^C^
		6.7 ± 1.2 ^a^	3.3 ± 0.75 ^b^	2.9 ± 0.50 ^b^
		3.3 ± 0.30 ^a^	2.3 ± 0.47 ^b^	3.1 ± 0.99 ^ab^
11	trans-cinnamic acid	0.80 ± 0.24 ^B^	0.79 ± 0.12 ^B^	2.4 ± 0.03 ^A^
		1.1 ± 0.11 ^b^	1.2 ± 0.20 ^b^	2.3 ± 0.59 ^a^
		1.5 ± 0.44 ^a^	1.1 ± 0.19 ^a^	1.3 ± 0.10 ^a^
12	gallocatechin	0.97 ± 0.01 ^B^	1.5 ± 0.11 ^A^	0.11 ± 0.01 ^C^
		3.5 ± 0.38 ^a^	3.2 ± 0.08 ^b^	0.41 ± 0.01 ^c^
		9.0 ± 0.70 ^a^	8.5 ± 1.04 ^a^	6.5 ± 0.36 ^b^
13	procyanidin B1	17 ± 1.45 ^B^	39 ± 2.3 ^A^	-
		161 ± 5.3 ^a^	102 ± 3.3 ^b^	11 ± 0.44 ^c^
		**-**	**-**	**-**
14	catechin	6.8 ±0.48 ^B^	12 ± 0.63 ^A^	0.78 ±0.06 ^C^
		24 ± 0.55 ^a^	18 ± 0.53 ^b^	4.8 ± 0.29 ^c^
		86 ± 3.7 ^a^	82 ± 5.5 ^ab^	74 ± 6.1 ^b^
15	procyanidin B2	0.60 ± 0.01 ^B^	0.90 ± 0.11 ^A^	-
		5.7 ± 0.29 ^a^	1.9 ± 0.22 ^b^	0.65 ± 0.07 ^c^
		6.6 ± 0. 21 ^a^	4.6 ± 0.41 ^c^	5.5 ± 0.4 ^b^
16	epicatechin	0.04 ± 0.00 ^B^	0.06 ± 0.02 ^A^	-
		0.68 ± 0.16 ^a^	0.43 ± 0.07 ^b^	-
		7.3 ± 0.52 ^a^	5.9 ± 0.55 ^b^	5.6 ± 0.18 ^b^
17	daidzin	-	-	-
		0.57 ± 0.05 ^a^	-	-
		3.8 ± 0.42 ^a^	3.0 ± 0.65 ^a^	3.5 ± 0.26 ^a^
18	rutin	18 ± 1.10 ^B^	54 ± 4.03 ^A^	14 ± 0.62 ^C^
		65 ± 3.37 ^a^	25 ± 0.68 ^b^	15 ± 0.10 ^c^
		0.63 ± 0.08 ^a^	0.70 ± 0.08 ^a^	0.39 ± 0.04 ^b^
20	vitexin	0.08 ± 0.03 ^B^	0.15 ± 0.03 ^A^	0.09 ± 0.02 ^C^
		0.13 ± 0.01 ^a^	0.06 ± 0.01 ^b^	0.03 ± 0.01 ^c^
		-	-	-
21	isoquercitrin	19 ± 0.11 ^B^	62 ± 2.8 ^A^	12 ± 0.56 ^C^
		177 ± 3.3 ^a^	108 ± 8.9 ^b^	53 ± 3.7 ^c^
		4.0 ± 0.24 ^b^	9.5 ± 1.1 ^a^	5.2 ± 0.36 ^b^
22	genistin	-	0.10 ± 0.01 ^B^	0.33 ± 0.19 ^A^
		0.46 ± 0.01 ^a^	0.18 ± 0.02 ^b^	0.11 ± 0.02 ^c^
		-	-	-
23	kaempferol-3-O-rutinoside	1.6 ± 0.13 ^C^	3.5 ± 0.34 ^A^	2.3 ± 0.10 ^B^
		4.4 ± 0.20 ^a^	1.5 ± 0.23 ^b^	1.0 ± 0.16 ^c^
		0.28 ± 0.00 ^a^	0.29 ± 0.01 ^a^	0.27 ± 0.01 ^a^
24	taxifolin	8.3 ± 0.27 ^B^	12 ± 0.64 ^A^	1.2 ± 0.10 ^C^
		19 ± 0.68 ^a^	8.6 ± 0.61 ^b^	3.2 ± 0.13 ^c^
		1.5 ± 0.16 ^b^	1.8 ± 0.02 ^a^	1.3 ± 0.02 ^b^
28	quercetin	2.3 ± 0.38 ^B^	6.5 ± 0.10 ^A^	-
		20 ± 0.67 ^a^	17 ± 0.89 ^b^	3.4 ± 0.10 ^c^
		0.14 ± 0.04 ^b^	0.40 ± 0.13 ^a^	0.13 ± 0.02 ^b^
32	phloroglucinol	10 ± 1.52 ^C^	24 ± 2.56 ^B^	36 ± 3.0 ^A^
		13 ± 1.24 ^c^	30 ± 2.32 ^a^	19 ± 3.7 ^b^
		42 ± 5.3 ^a^	36 ± 5.0 ^b^	28 ± 5.1 ^c^
33	3,4-dihydroxyphenylacetic acid	-	-	-
		-	-	-
		17 ± 0.56 ^a^	18 ± 1.5 ^a^	18 ± 0.40 ^a^
	Total	100 ± 7.0 ^B^	241 ± 17 ^A^	88 ± 6.2 ^B^
		532 ± 19 ^a^	333 ± 20 ^b^	136 ± 10 ^c^
		296 ± 19 ^a^	276 ± 26 ^a^	253 ± 21 ^a^

- not detected. OJ: Oral digestive fluid; OF: free phenol from oral residue; OB: oral residue bound phenol; GJ: gastric digestive fluid; GF: gastric residue free phenol; GB: gastric residue bound phenol; IJ: small intestinal digestive fluid; IF: small intestinal residue free phenol; IB: small intestinal residue bound phenol. A–B (a–c): Different letters indicate significant differences in polyphenol content among different digestion stages, uppercase letters represent total phenols(A), lowercase letters represent free or bound phenols(a), *p* < 0.05.

## Data Availability

The original contributions presented in the study are included in the article/Supplementary Materials; further inquiries can be directed to the corresponding authors.

## Supplementary Materials

The following supporting information can be downloaded at: https://www.mdpi.com/article/10.3390/foods15111985/s1, Figure S1: Antioxidant capacity at each digestive stage.
