# Supplementary material for: Bioaccessibility and Dynamic Changes in Free and Bound Phenolics in Rice Bean (Vigna umbellata) During Simulated Digestion
Source: Foods. 2026 Jun 3;15(11):1985. doi: 10.3390/foods15111985 (PMC13257373; doi:10.3390/foods15111985)
Supplement: Supplementary file 1 [file foods-15-01985-s001.zip › foods-4325145-supplementary.pdf]

Supplementary Materials: Antioxidant activity and correlation analysis

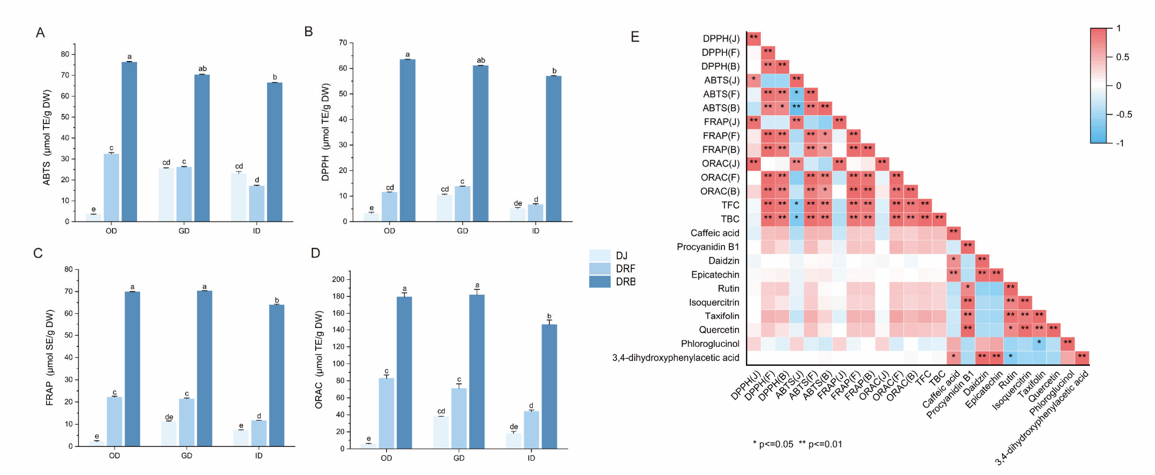

Figure S1 Antioxidant capacity at each digestive stage. (A) ABTS: free radical scavenging ability; (B) DPPH: free radical scavenging ability; (C) FRAP: antioxidant activity; (D) ORAC: antioxidant activity;(E) Correlation between Polyphenols and Antioxidant Activity during Digestion; DJ: digestive juice; DRF: free phenols in the digestion residue.; DRB: bound phenols in the digestion residue.
